# Comorbidity in the aging population with multiple sclerosis: a Danish nationwide study

**DOI:** 10.3389/fneur.2023.1297709

**Published:** 2023-11-24

**Authors:** Rolf Pringler Holm, Malthe Faurschou Wandall-Holm, Finn Sellebjerg, Melinda Magyari

**Affiliations:** ^1^Danish Multiple Sclerosis Registry, Department of Neurology, Copenhagen University Hospital–Rigshospitalet, Glostrup, Denmark; ^2^Danish Multiple Sclerosis Center, Department of Neurology, Copenhagen University Hospital–Rigshospitalet, Glostrup, Denmark

**Keywords:** multiple sclerosis, aging, comorbidity, hospital admissions, mortality, patient-centered care

## Abstract

**Introduction:**

Multiple sclerosis (MS) is a neurodegenerative disease accumulating disabilities over time. However, the mean age of individuals with MS is increasing, consequently elevating their risk of developing comorbidities. Comorbidities' impact on MS is widely debated. Yet very few countries possess population-based registries, which provide unique opportunities for individual-level data linkage. This study aims to assess acute and chronic comorbidities among elderly patients with MS, comparing them to matched controls. Additionally, this study seeks to investigate the influence of chronic comorbidities on all-cause mortality.

**Methods:**

A nationwide register-based study using the Danish Multiple Sclerosis Registry to identify all living patients with MS older than 50 years at the reference date (January 1st, 2022). Patients were matched 1:10 with individuals from the general population. Comprehensive healthcare data within the Danish hospital system were obtained. Chronic comorbidities were classified according to the Charlson Comorbidity Index, while acute comorbidities were based on ICD-10 codes and an “acute” admission type. To investigate all-cause mortality, a Cox regression analysis was conducted.

**Results:**

The study encompassed a total of 8,688 individuals with MS, matched with 86,880 controls. The mean age was 63.5 years, with females constituting 68.3%. Individuals with MS exhibited a higher frequency of acute hospitalizations (OR: 2.1, 95% CI: 1.9–2.2), primarily due to various infectious diseases, along with longer median hospital stays (4 vs. 3 days, *p* < 0.001). When assessed using the Charlson Comorbidity Index, individuals with MS carried a significantly greater burden of chronic comorbidities (*p* < 0.001). The most prevalent chronic comorbidity among individuals with MS was “Uncomplicated Diabetes” (20.1%). Notably, while individuals with MS displayed an overall lower 5-year survival rate, this difference ceased to be statistically significant among those with a high Charlson Comorbidity Index score of ≥4 (*p* = 0.32).

**Conclusion:**

This study highlights a heightened prevalence of both acute and chronic comorbidities among individuals with MS, with chronic comorbidities significantly increasing the risk of mortality. These findings underscore the critical importance of factoring in comorbidities when devising treatment strategies for individuals living with MS.

## 1 Introduction

In recent decades, there has been an increase in the mean age of people with multiple sclerosis (MS), attributed to various contributing factors (1, 2). These factors include the growing incidence of late-onset MS, characterized by the manifestation of initial clinical symptoms after the age of 50 years (1, 2). Additionally, advancements in disease-modifying therapies (DMTs), improved supportive care, developments in diagnostics, and a general rise in life expectancy across the population have played significant roles. However, it is crucial to recognize that advanced age also serves as a risk factor for comorbidities.

Considering that nearly half of the Danish MS population is older than 50 years (median age: 54.9, SD: 14.5, data from the Danish Multiple Sclerosis Registry), it has become important to explore the prevalence and impact of comorbidities on mortality within elderly people with MS. The investigation of comorbidities in people with MS has gained substantial attention in recent years due to its influence on disease activity and other clinical outcomes (3–5). Moreover, a higher burden of concurrent diseases often correlates with poorer prognostic outcomes, and comorbidities contribute to delays in MS diagnosis and can impact the initiation of DMTs (6–9).

The specific characteristics and challenges faced by elderly individuals with MS and comorbidities remain inadequately described. This is primarily due to the exclusion of people older than 55 years in many clinical trials. Furthermore, substantial comorbidities often serve as exclusion criteria, making this group of people more complex to manage and treat within a clinical setting. The issue is further aggravated by the increased risk of polypharmacy with increased concurrent disease conditions, adding further complexity to treatment decisions for these patients.

Although previous studies have reported an elevated risk of specific comorbidities in people with MS, no study has yet investigated the prevalence of the most common acute and chronic comorbidities within the aging Danish MS population. Obtaining such knowledge could facilitate the targeted allocation of resources, allowing for the implementation of prevention strategies tailored to high-risk patients.

The objective of this registry-based study was to investigate the prevalence of common acute and chronic comorbidities among contemporary elderly people with MS, comparing them to matched controls from the general population. Additionally, we aimed to investigate the potential influence of chronic comorbidity on all-cause mortality within this population.

## 2 Methods

### 2.1 Study design and study population

This study is a nationwide population-based study conducted in Denmark. All people with a diagnosis of MS were identified from the Danish Multiple Sclerosis Registry (DMSR) (10). To be eligible for inclusion, people with MS had to meet the following criteria: age above 50 years, residency in Denmark, and alive at the reference date (January 1st, 2022). A 25% random sample of the general Danish population, excluding individuals with MS, was used to select 10 matched controls for each patient. Matching was based on sex, exact age, ethnicity, and geographical region at the reference date. Ethnicity was categorized as immigrants (if people were born outside of Denmark) and descendants (if people were born in Denmark, but both parents were born outside of Denmark) or Danish (all others).

### 2.2 Data sources and variables

The utilization of the unique personal identification number, assigned to all Danish citizens and individuals with a permanent address residing in the country, enabled cross-linkage between national registries at the individual level (11).

#### 2.2.1 Clinical data on MS

The DMSR is a comprehensive nationwide population-based registry that has been collecting data on all people with MS since 1956. Currently, information is sourced from the 13 MS clinics dispersed throughout the country and is directly entered by clinicians during clinical visits into an online data collection platform. Following the introduction of DMTs in 1996, data entry for treated patients became mandatory. The registry serves as the foundation for national clinical quality indicators, ensuring a high degree of completeness and data validity (12). The DMSR encompasses clinical data on basic personal information, diagnostics, phenotypes, disability status, treatments, imaging, and more.

From the DMSR, we extracted several key variables including age, sex, age at MS onset, onset symptoms, current phenotype, the most recently recorded Expanded Disability Status Scale (EDSS) score within the past 2 years, duration since the last EDSS score record, MRI and clinical visit information, relapse activity, and visit frequency. Disease duration was calculated as the difference in years between the onset of MS and the reference date. Additionally, we created a binary variable termed “lost clinical contact”, indicating whether patients had no recorded clinical visit in an MS clinic, EDSS score, MS-related treatment, or MS-related MRI within the last 10 years. However, it is crucial to note that these patients might have had interactions with the healthcare system concerning other chronic or acute diseases.

#### 2.2.2 Comorbidity

The Danish National Patient Registry (DNPR) contains information on admissions to somatic hospital departments since 1977 with the addition of emergency departments and psychiatric departments in 1995 (13). The DNPR uses a Danish adaptation of the coding system “International Classification of Diseases, 10th revision” (ICD-10).

From the DNPR we collected the number of acute hospital admissions in 2021, duration of stay, and the five most overall frequent diagnoses related to acute admission in both people with MS and controls from the general population, allowing overlap. To detect the presence of chronic comorbidities, we searched the last 10 years for diagnosis codes consistent with a disease listed in the Charlson Comorbidity Index or a psychiatric disorder as previously categorized in MS literature (14, 15). The full list of used ICD-10 codes is available in the referenced articles (14, 15).

### 2.3 Statistical analysis

The characteristics of the people with MS and controls from the general population are presented as frequencies with corresponding percentages for categorical variables, while continuous variables are reported as mean values with standard deviation (SD) or as median values with the 1st and 3rd quartiles.

All statistical comparisons were performed between the two groups (MS or general populations) accounting for the clustering effect of the matched study design. For binary outcomes, odds ratios (OR) with corresponding 95% confidence intervals (95% CI) and *p*-values were calculated using a generalized estimating equation with a logit link function. The model was adjusted for the clustering of individuals into matched groups, assuming an independent correlation structure within clusters. Exponentiated estimates and joint confidence limits were calculated for the primary predictor. For ordinal outcomes, *p*-values were assessed using a generalized linear model with a multinomial distribution and a cumulative logit link function. The model accounted for the clustering of individuals within matched groups by introducing a subject-specific random effect, assuming an independent correlation structure within clusters. *P*-values for the significance of the association were calculated using a joint test. The rate ratio (RR) with the corresponding 95% CI and *p*-value of acute admission rates was calculated using a generalized linear model with a negative binomial distribution to adjust for overdispersion. The model further accounted for clustering in the dataset by utilizing cluster-robust variance-covariance matrices. The rate ratio and its 95% CI were computed based on the model coefficient and cluster-robust standard errors. To assess differences in length of stay of acute admissions we used a mixed-effects model. The model included a fixed effect group status (case or control) and introduced a random effect for the matched groups to capture the intra-cluster correlation. The empirical variance estimator was used to obtain root-unbiased standard errors.

To assess all-cause mortality, we did a Cox regression analysis without competing risks. We followed participants from January 1st, 2016, until either the event of interest (death from all causes) or censoring [study end (December 31st, 2021) or emigration]. We calculated the Charlson Comorbidity Index score (CCI score) by looking at the previous 4 years (2012–2015) in the DNPR for the presence of an ICD-10 code fulfilling the criteria (14).

We categorized CCI scores into three groups: none or mild comorbidity (0–1), moderate comorbidity (2–3), and high comorbidity (≥4). We used group (MS or general population) and comorbidity categories and added an interaction term to determine whether the effect of comorbidities on mortality differed in the two groups. We accounted for the clustered data by using “the robust sandwich estimator”. By using the CCI groups, we were able to create measures reflecting a pooled analysis considering all chronic diseases together to elucidate the overall difference between people with MS and the general population.

Data management, statistical analyses, and visualizations were conducted using SAS version 9.4 (SAS Institute Inc., Cary, NC, USA).

### 2.4 Ethics, approvals, and data access

Obtaining informed consent or ethical approval is not mandatory for observational register-based studies in Denmark. This study adheres to the Danish General Data Protection Regulation (GDPR) and is registered with the Knowledge Center for Data Reviews, which serves as the data-responsible entity approved by the Danish Data Protection Agency. Access to the data is available upon submission of a qualified request. To ensure confidentiality, any cells containing information from fewer than three subjects (or neighboring cells enabling cross-cell calculations) are censored to prevent personally identifiable data. The preparation of data was conducted on secure servers provided by Statistics Denmark with the Approved Journal Number 10.123.

## 3 Results

**Figure 1:**
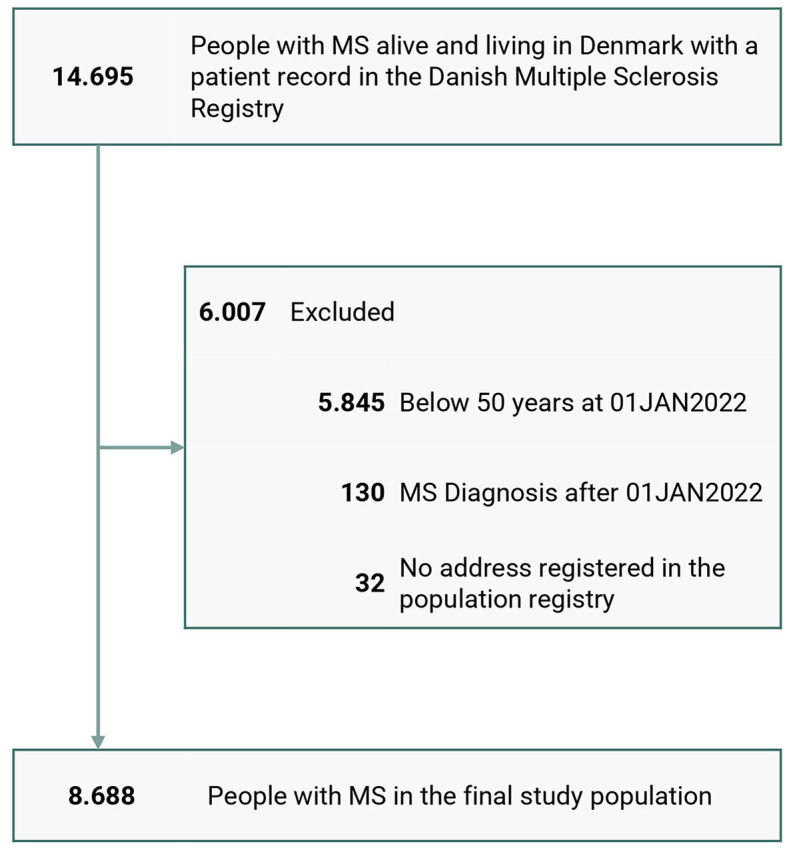
Disposition chart.

Figure 1 shows the disposition chart, and the baseline characteristics of the MS population are presented in Table 1. The study population consisted of 8,688 people with MS and 86,880 matched individuals from the general population. The mean age of the entire population was 63.5 years (SD: 9.0) at the reference date (January 1st, 2022). Of all the included individuals 99.2% had Danish ethnicity. The female-male ratio was 2:1 (68.3% females), and the people with MS had a mean age of 39.3 years (SD: 11.1) at clinical onset, and a mean disease duration of 24.2 years (SD: 12.4) at the reference date. The most frequent onset symptoms were “Sensory” (28.7%), “Pyramidal” (18.0%), and “Optic nerve” (15.6%). The distribution of phenotypes at the reference date among the people with MS was 42.1% RRMS, 25.9% SPMS, 14.2% PPMS, and 17.8% unclassified. The latest EDSS score within 2 years from the reference date had a median of 3.5 (Q_1_-Q_3_ = 2.0–6.0) and only 409 (4.7%) had one or more relapses in the last 2 years. The median time since the last visit was 1.1 years (Q_1_-Q_3_ = 0.5–9.7). In total 20.9% of the living people with MS older than 50 years were defined as having “lost clinical contact” with the MS clinics.

**Table 1:** Demographic and clinical characteristics of the MS population.

	**Multiple sclerosis**
Number of patients	8.688
Age, years, mean (SD)	63.5 (9.0)
Age at onset, years, mean (SD)	39.3 (11.1)
Disease duration, years, mean (SD)	24.2 (12.4)
**Sex, No. (%)**	
Male	2.751 (31.7)
Female	5.937 (68.3)
**Ethnicity**,^a^ **No. (%)**	
Danish	8.617 (99.2)
Other	71 (0.8)
**Onset symptoms, No. (%)**	
Brainstem	560 (6.4)
Cerebellar	299 (3.4)
Optic nerve	1.353 (15.6)
Pyramidal	1.561 (18.0)
Sensory	2.493 (28.7)
Sphincter	88 (1.0)
Multifocal	1.158 (13.3)
Other	310 (3.6)
Missing	866 (10.0)
**Phenotype, No. (%)**	
RRMS	3.661 (42.1)
SPMS	2.254 (25.9)
PPMS	1.230 (14.2)
Unspecified	1.543 (17.8)
**EDSS**	
Latest EDSS within two years, median (Q_1_-Q_3_), *n*_miss_	3.5 (2.0–6.0), 3.677
Time since last EDSS, years, median (Q_1_-Q_3_), *n*_miss_	0.8 (0.4–1.9), 2.048
**One or more relapses in the last 2 years, No. (%)**	
Yes	409 (4.7)
No	8.279 (95.3)
**MRI**	
Time since last MRI, years, median (Q_1_-Q_3_), *n*_miss_	1.0 (0.4–3.0), 3.424
Time since last visit, years, median (Q_1_-Q_3_), *n*_miss_	1.1 (0.5–9.7), 0
**One or more visits in the last 2 years, No. (%)**	
Yes	5.193 (59.8)
No	3.495 (40.2)
**Lost clinical contact**,^b^ **No. (%)**	
Yes	1.816 (20.9)
No	6.872 (79.1)

^a^Ethnicity was categorized as immigrants (if born outside of Denmark) and descendants (if born in Denmark, but both parents born outside of Denmark) or Danish (all others).

^b^No recordings in the last 10 years of a visit to an MS clinic, an EDSS score, a DMT, or an MS-related MRI.

RRMS, relapsing-remitting multiple sclerosis; SPMS, secondary progressive multiple sclerosis; PPMS, primary progressive multiple sclerosis; EDSS, Expanded Disability Status Scale; SD, standard deviation; Q_1_-Q_3_, first and third quartile; N_miss_, number of missing; MRI, magnetic resonance imaging.

### 3.1 Acute hospital admissions

Table 2 presents the details of acute hospital admissions during 2021. A statistically significant difference was observed in the proportion of people with MS experiencing one or more acute hospital admissions compared to the matched controls from the general population (OR: 2.1, 95% CI: 1.9–2.2). Furthermore, the difference in proportions increased with the number of acute hospital admissions when subdivided into 1, 2, or ≥3 admissions. The calculated rate ratio for acute hospital admission was 2.2 (95% CI: 2.1–2.4). These results represent aggregated outcomes analyzed collectively to assess the overall differences between individuals with MS and the general population.

**Table 2:** Acute hospital admissions in the year 2021.

	**General population**	**Multiple sclerosis**	***p*-value**	**Estimates (95% CI)**
Number of patients	86.880	8.688		
One or more admissions in 2021, No. (%)^a^	6.784 (7.8)	1.300 (15.0)	< 0.001	OR: 2.1 (1.9–2.2)
If yes, length of stay, days, median (Q_1_-Q_3_)	3 (1–7)	4 (1–9)	< 0.001	
**Number of acute hospital admissions, No. (%)**				
0	80.086 (92.2)	7.388 (85.0)	< 0.001	
1	4.515 (5.2)	745 (8.6)		
2	1.291 (1.5)	268 (3.1)		
≥3	978 (1.1)	287 (3.3)		
Rate of acute hospital admissions	0.13	0.29	< 0.001	RR: 2.2 (2.1–2.4)
**Acute admission diagnoses, No. (%)**				
Pneumonia/DJ189	278 (0.3)	92 (1.1)	< 0.001	OR: 3.3 (2.6–4.2)
Bacterial infection, unspecified/DA499	89 (0.1)	60 (0.7)	< 0.001	OR: 6.8 (4.9–9.4)
Acute abdominal pain/DR100	192 (0.2)	20 (0.2)	0.86	OR: 1.0 (0.7–1.7)
Unspecified disorder/DZ039	271 (0.3)	70 (0.8)	< 0.001	OR: 2.6 (2.0–3.4)
Urinary infection/DN390	174 (0.2)	177 (2.0)	< 0.001	OR: 10.4 (8.4–12.8)
Urosepsis/DA419B	63 (0.1)	78 (0.9)	< 0.001	OR: 12.5 (9.0–17.3)
Ischemic stroke/DI639	187 (0.2)	24 (0.3)	0.24	OR: 1.3 (0.8–2.0)

^a^Requiring a stay of at least 1 day.

RR, rate-ratio; OR, odds ratio; CI, confidence interval; Q_1_-Q_3_, first and third quartile.

Among people with MS, “urinary infection” was the most frequent reason for acute hospital admission, with a statistically significantly higher incidence compared to the control group (OR: 10.4, 95% CI: 8.4–12.8). A similar pattern was observed for “urosepsis”, albeit with a lower incidence (OR: 12.5, 95% CI: 9.0–17.3). Additionally, the incidence of “pneumonia,” “bacterial infection, unspecified,” and “unspecified disorder” differed between the MS population and the control group, although these differences were less pronounced (OR: 2.6–6.8). No statistically significant difference between the two groups was found when comparing the incidence of “acute abdominal pain” (OR: 1.0, 95% CI: 0.7–1.7) and “ischemic stroke” (OR: 1.3, 95% CI: 0.8–2.0).

The duration of hospital stays for admitted individuals was both clinically and statistically significantly different in the two groups, with a median of 4 days (Q_1_-Q_3_ = 1–9) among people with MS and 3 days (Q_1_-Q_3_ = 1–7) among the control group (*p* < 0.001).

### 3.2 Chronic comorbidities

The results of chronic comorbidities used in the Charlson Comorbidity Index and psychiatric diseases are presented in Tables 3, 4, respectively. Upon investigating the comorbidity burden (CCI scores) within the study population, we observed an overall significant difference in favor of the control group when comparing the two groups.

**Table 3:** Chronic diseases according to the Charlson Comorbidity Index, last 10 years.

	**General population**	**Multiple sclerosis**	***p*-value**	**Estimates (95% CI)**
Number of patients	86.880	8.688		
**Disease, No. (%)**				
Myocardial infarction	1.788 (2.1)	185 (2.1)	0.65	OR: 1.0 (0.9–1.2)
Congestive heart failure	1.767 (2.0)	148 (1.7)	0.04	OR: 0.8 (0.7–1.0)^a^
Peripheral vascular disease	2.024 (2.3)	207 (2.4)	0.75	OR: 1.0 (0.9–1.2)
Cerebrovascular disease	4.067 (4.7)	514 (5.9)	< 0.001	OR: 1.3 (1.2–1.4)
Dementia	798 (0.9)	144 (1.7)	< 0.001	OR: 1.8 (1.5–2.2)
Chronic pulmonary disease	5.161 (5.9)	446 (5.1)	< 0.001	OR: 0.9 (0.8–0.9)
Rheumatic disease	1.995 (2.3)	150 (1.7)	< 0.001	OR: 0.7 (0.6–0.9)
Peptic ulcer	815 (0.9)	108 (1.2)	0.01	OR 1.3 (1.1–1.6)
Liver disease, mild	1.141 (1.3)	101 (1.2)	0.24	OR: 0.9 (0.7–1.1)
Diabetes, uncomplicated	14.086 (16.2)	1.744 (20.1)	< 0.001	OR: 1.3 (1.2–1.4)
Renal disease, mild to moderate	810 (0.9)	105 (1.2)	0.01	OR: 1.3 (1.1–1.6)
Diabetes, complicated	6.960 (8.0)	766 (8.8)	0.01	OR: 1.1 (1.0–1.2)^a^
Hemiplegia or paraplegia	210 (0.2)	223 (2.6)	< 0.001	OR: 10.9 (9.0–13.2)
Any malignancy	6.852 (7.9)	620 (7.1)	0.01	OR: 0.9 (0.8–1.0)^a^
Liver disease, moderate to severe	164 (0.2)	12 (0.1)	0.29	OR: 0.7 (0.4–1.3)
Renal disease, severe	313 (0.4)	20 (0.2)	0.05	OR: 0.6 (0.4–1.0)
HIV infection	16 (0.0)	0 (0.0)	-	-
Metastatic solid tumor	601 (0.7)	59 (0.7)	0.89	OR: 1.0 (0.7–1.3)
AIDS	3 (0.0)	0 (0.0)	-	-
**CCI categories, No. (%)**				
0–1: No comorbidity or one low risk	69.132 (79.6)	6.687 (77.0)	< 0.001	
2–3: Several low-risk or one high-risk comorbidity	14.455 (16.6)	1.651 (19.0)		
≥4: Many and/or high-risk comorbidities	3.293 (3.8)	350 (4.0)		

^a^In cases of significant p-values and confidence intervals including 1, rounding is the reason.

CCI, Charlson Comorbidity Index; OR, odds ratio; CI, confidence interval.

**Table 4:** Psychiatric diseases, last 10 years.

	**General population**	**Multiple sclerosis**	***p*-value**	**Estimates (95% CI)**
Number of patients	86.880	8.688		
**Disease, No. (%)**				
Depression	1.434 (1.7)	142 (1.6)	0.91	1.0 (0.8–1.2)
Anxiety	664 (0.8)	50 (0.6)	0.05	0.8 (0.6–1.0)
Bipolar disorder	292 (0.3)	31 (0.4)	0.75	1.1 (0.7–1.5)

OR, odds ratio; CI, confidence interval.

There was no elevated risk of registered “depression,” “anxiety,” or “bipolar disorder” among elderly people with MS compared to the control group with people from the general population, see Table 4.

### 3.3 All-cause mortality

The interaction term between the group definition (MS or general population) and comorbidity category was highly statistically significant (*p* < 0.001) and included in the final model; test results are presented in Table 5.

**Table 5:** Likelihood-ratio tests, all-cause mortality.

**Type 3 tests**	***p*-value**
Group (multiple sclerosis vs. general population)	< 0.001
Comorbidity category	< 0.001
Interaction between group and comorbidity category	< 0.001

**Figure 2:**
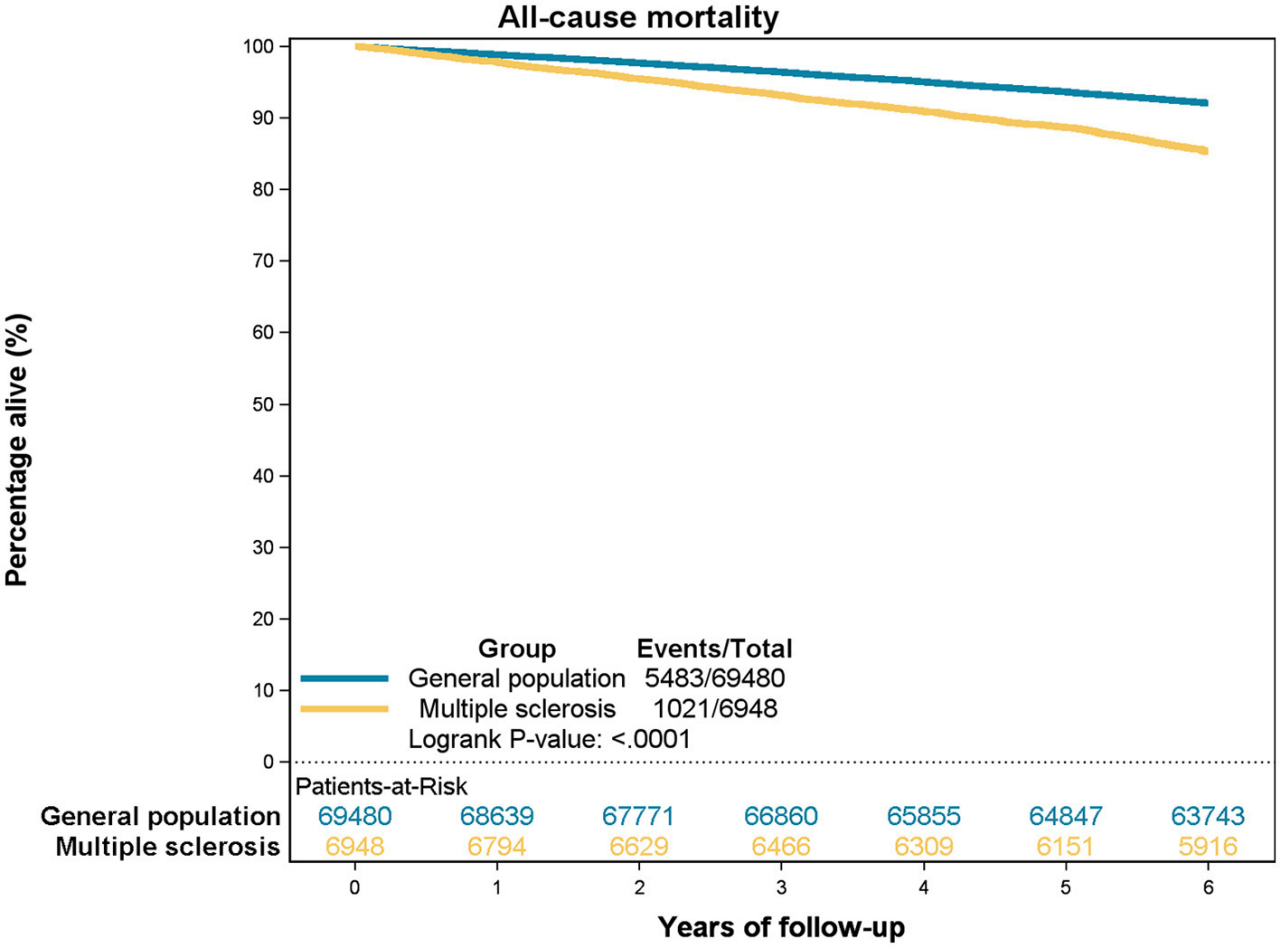
All-cause mortality, multiple sclerosis vs. general population, all.

**Figure 3:**
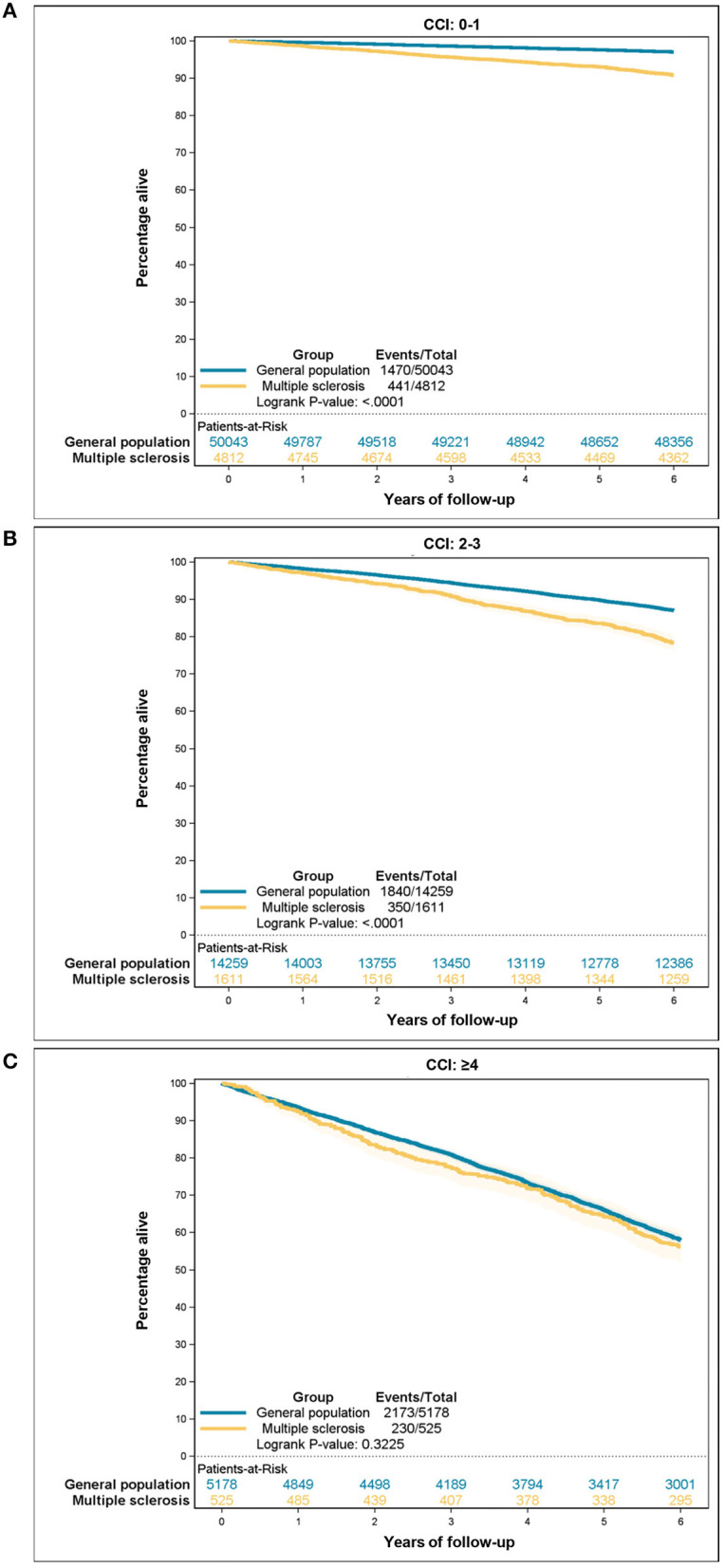
All-cause mortality according to the Charlson Comorbidity Index (CCI) category: **(A)** CCI 0–1, **(B)** CCI 2–3, **(C)** CCI ≥ 4.

Table 6 presents the estimates for all-cause mortality risks according to the comorbidity categories among people with MS and the control group. The CCI score was positively correlated with an increased hazard ratio (HR) for death in both populations. However, people with MS had higher HR in all three comorbidity categories, though the difference decreased with CCI ≥ 4. Additionally, a higher CCI score corresponded to a lower 5-year survival for both groups. Nevertheless, people with MS experienced worse overall 5-year survival than the general population (88.7 vs. 93.6%). When the two groups were stratified by comorbidity categories the reduced survival among people with MS remained statistically significant among CCI score 0–1 and CCI score 2–3, but this difference was no longer statistically significant among CCI score ≥4. Survival curves are illustrated in Figures 2, 3A–C.

**Table 6:** Estimates, all-cause mortality.

	**Hazard ratio**	**95% CI**
**Multiple sclerosis**		
CCI 0–1	3.2	2.9–3.6
CCI 2–3	8.1	7.2–9.2
CCI ≥ 4	19.3	16.8–22.3
**General population**		
CCI 0–1	1 (ref.)	1 (ref.)
CCI 2–3	4.6	4.3–5.0
CCI ≥ 4	18.0	16.8–19.4
**5-year survival**	**Percentage survived**	**95% CI**
**Overall**		
General population	93.6	93.5–93.8
Multiple sclerosis	88.7	87.9–89.4
**CCI 0–1**		
General population	97.6	97.5–97.7
Multiple sclerosis	93.0	92.3–93.8
**CCI 2–3**		
General population	89.8	89.3–90.3
Multiple sclerosis	83.5	81.7–85.4
**CCI** ≥**4**		
General population	66.1	64.8–67.4
Multiple sclerosis	64.4	60.4–68.6

CCI, Charlson Comorbidity Index; CI, confidence interval; Ref., reference group.

## 4 Discussion

This population-based study conducted with nationwide registry data from Denmark revealed significant variations in the prevalence of both acute and chronic comorbidities among older people with MS, as compared to matched controls from the general population.

### 4.1 Acute hospital admissions

The results revealing the statistically significant disparity in the frequency of acute hospital admissions indicate an increased burden on the acute healthcare system among people with MS.

Our findings reveal a 10-fold increase in the incidence of urinary tract infections and cases of urosepsis among people with MS. This pronounced difference can be attributed to the well-documented association between MS and bladder dysfunction affecting up to 70% of people with MS, which significantly elevates the susceptibility to urinary tract infections (16, 17). Admission due to pneumonia was also more than three times as frequent among people with MS than in the controls from the general population. This aligns with a study from 2009 that investigated risk factors for pneumonia and found MS to be the strongest (higher than diabetes and chronic respiratory diseases) (18). Our findings also showed an elevated frequency of admitted patients due to bacterial infections like previous studies reporting elevated risk of infections in general among people with MS (19–21).

Thus, our findings are consistent with prior literature demonstrating an elevated risk of various acute infections among individuals with MS. Early detection in the primary health sector resulting in earlier treatment or other prevention strategies might decrease the burden on the acute healthcare system and thereby potentially save time and money (22).

### 4.2 Chronic comorbidities

Generally, the literature concerning chronic comorbidities in individuals with MS reveal inconsistent findings, presumably due to different case definition and data sources. To enhance clarity, we decided to only report a subset of disease categories in the subsequent discussion, comparing them with the existing literature.

#### 4.2.1 Vascular comorbidities

Our investigation into vascular comorbidities yielded diverse findings. We observed no differences in the prevalence of myocardial infarction or peripheral vascular disease, and a lower prevalence of congestive heart failure, while cerebrovascular disease was more common among people with MS.

We found three studies supporting our results regarding ischemic heart disease, while two other studies and a comprehensive review article all reported an elevated risk of ischemic heart disease (23–28). Three studies and a meta-analysis, including results from a Danish study, reported an elevated risk of cerebrovascular events as observed in the present study, but when one of them excluded the first year of follow-up to avoid ascertainment bias, the elevated risk was no longer statistically significant (24, 25, 27–29). Furthermore, when the British study conducted by Chou et al. subdivided cerebrovascular events in “before” and “after” MS diagnosis, the significant difference was only seen “before” MS diagnosis (25). Misclassified white matter lesions on MRI or relapses mistaken for cerebrovascular insult before MS diagnosis might explain this finding.

#### 4.2.2 Diabetes

Diabetes was the most common chronic comorbidity in our study population. We found a statistically higher prevalence of diabetes among people with MS regardless of whether the condition was with or without complications. Most of the previous studies support our results, but some still contradict them (25, 30–33). The association between MS and diabetes may arise from shared disease mechanisms, genetic factors, or lifestyle changes prompted by an MS diagnosis. Regardless of the underlying cause, it remains crucial to equip healthcare providers with the tools to effectively handle individuals facing both conditions.

#### 4.2.3 Cancer

When investigating cancer, our study did not demonstrate an increased prevalence among individuals with MS. Our results showed slightly lower odds for “any malignancy” among people with MS. However, it is essential to interpret these findings cautiously, considering the complex and evolving nature of the relationship between MS and cancer, which has yielded conflicting results in previous studies. Six studies supported our finding of no increased risk of cancer, while others in part or completely contradict our findings (34–41).

#### 4.2.4 Neurological and psychiatric diseases

Our study revealed a higher prevalence of hemiplegia and paraplegia among people with MS. Similarly, dementia without specification was more common in the MS population. However, both diagnoses likely refer to MS symptoms rather than distinct comorbidities.

Unexpectedly we observed a noticeably lower prevalence of psychiatric diseases compared to previously reported rates in MS populations, and we found no differences between our MS cohort and controls from the general population (25, 42, 43). Differences in prevalence observed in other studies may be attributed, at least in part, to the countries investigated. For instance, previous reviews have revealed lower depression prevalence in Europe compared to North America (42, 43). Another potential contributing factor could be the increased incidence and prevalence of depression observed among younger individuals, which would not be observed in our study population consisting of people above 50 years of age (44, 45).

Furthermore, we used diagnoses from the DNPR, which are registered during hospital admissions, which may have led to potential underestimation of psychiatric diagnoses, as most individuals with mild depression or mild anxiety are treated by their general practitioners. Patients admitted to the hospital receive a primary diagnosis code that corresponds to the main reason for their admission. However, it is important to note that additional diagnosis codes may also be assigned, reflecting pre-existing conditions or comorbidities that are relevant to the patient's overall health management, even if they were not the primary reason for the current admission. For instance, if a patient has a previous diagnosis of depression from their general practitioner but has not been admitted to the hospital specifically for depression, the relevant diagnosis code for depression may still be included when they are admitted for a different condition, such as MS, to ensure comprehensive and informed healthcare delivery. We believe that the potential underestimation of psychiatric diseases equally affects both people with MS and controls from the general population. Alternatively, there could be a bias toward relatively higher detection rates among people with MS due to more frequent outpatient hospital visits.

#### 4.2.5 Renal disease

Our study revealed ambiguous results regarding renal disease as people with MS were more likely to have “mild to moderate renal disease”, while “severe renal disease” was less frequent with a borderline statistical significance (*p* = 0.05).

A review published in 2015 including 6 articles with data on renal disease from 1989 to 2009 reported a prevalence of “renal disease” of 0.74–2.49% and “renal failure” of 0–0.78% among people with MS (46). Only three of the studies included in the review compared people with MS to background populations: two found no difference and one found renal failure to be less common in the MS population (the last-mentioned study was done in 1994 on patients aged ≥65 years). These results were supported by a study from 2019 reporting a hazard ratio of “renal disease” to be 0.9 (95% CI: 0.58–1.38) among people with MS compared to matched controls (25).

Our results might be influenced by closer monitoring of people with MS compared to the general population, explaining the higher number of “mild to moderate” cases because these cases do not necessarily bring otherwise healthy people to the doctor. At the same time, early detection gives the opportunity the intervene and maybe prevent escalation to “severe” disease.

#### 4.2.6 Abdominal diseases

We found no differences regarding liver diseases between people with MS and controls from the general population. This aligns with the existing literature despite the potential liver-related side effects associated with all MS treatments and a suggested association between autoimmune liver diseases and MS (25, 46–49). In terms of peptic ulcer, our results showed a slightly higher prevalence among people with MS, which is partly supported by the literature, despite high heterogeneity in results (25, 46, 47). The use of glucocorticoid treatment in MS might have had some influence on this finding.

#### 4.2.7 All-cause mortality

We found increased all-cause mortality among people with MS compared to controls from the general population, which is in line with previously reported findings (25). Furthermore, it has been shown that having comorbidities before MS diagnosis increases all-course mortality among people with MS (25). We are, to our knowledge, the first to contribute with knowledge about the relative impact of comorbidity burden on all-course mortality among people with MS. Our findings reveal that coexisting MS at first increases all-course mortality, but when the comorbidity burden gets high enough (CCI score ≥4) people with MS have the same hazard of death as matched controls from the general population.

### 4.3 General considerations

Certainly, comparing results across different studies that investigate comorbidities can raise difficulties due to a range of factors, including the lack of standardized approaches to categorization and presentation results, as well as differences in study design, populations, data sources, and analytical methods. In the following, we will elaborate further.

#### 4.3.1 Categorization

Categorizing and presenting results related to comorbidities poses a challenge due to the extensive array of diagnoses, which is further complicated by the divergence in terminology (such as ICD-10, MedDRA, etc.) employed across various countries. Given the myriad of diagnoses, researchers often resort to categorizing comorbidities, albeit through varying approaches, which makes direct comparisons difficult. In this study, we opted to employ the internationally recognized Charlson Comorbidity Index, a tool designed to prognosticate the impact of comorbidities, which aligned with our research objectives (50). Additionally, this index is compatible with ICD-10 codes, the coding system utilized within the Danish healthcare system (14). Importantly, the index does not directly include MS, facilitating a direct comparison of CCI scores between individuals with MS and controls without necessitating alterations to the index. This was pivotal as our investigation aimed to elucidate how the burden of comorbidities influences life expectancy in individuals with MS relative to control subjects.

To illustrate the lack of standardized data collection and presentation, our literature search revealed different approaches to categorization and the resulting impact on findings. Despite the common data source, two British studies reported different results due to variations in their categorization and presentation of comorbidities. Palladino et al. reported an elevated risk of “cardiovascular disease” among people with MS, while Choi et al. found no elevated risk of “myocardial infarction” before or after MS diagnosis (25, 28). These discrepancies highlight the challenges posed by non-standardized approaches to categorization and presentation and emphasize the need for a more unified approach in comorbidity research to facilitate more accurate comparisons.

#### 4.3.2 Study population

We chose to focus on comorbidities among older people with MS due to the increased risk of comorbidities with age. Additionally, the elderly MS population is growing, making it increasingly relevant to examine their comorbidities. Including younger patients would probably have resulted in a lower prevalence of comorbidities in both groups, making it more difficult to detect meaningful differences.

Furthermore, the interplay between biological sex, age, and comorbidities presents a multifaceted analytical challenge. Our study did not disaggregate data by sex within different age groups. We prioritized providing a comprehensive overview of comorbidities across the older MS population, rather than conducting a granular analysis by sex and age categories. Nonetheless, we acknowledge the potential impact of these variables and suggest that a detailed analysis that considers these stratifications could be highly informative. This more focused approach represents a valuable direction for future research.

#### 4.3.3 The strength of the data source

In Denmark, we are fortunate to have a large number of nationwide registers with high data quality, which reduces the risk of selection bias. Furthermore, unique social security numbers (CPR numbers) make it possible to link data on the individual level. Many epidemiological studies utilize administrative health care or insurance data, which may not be as comprehensive or accurate as national disease registries. Additionally, most previous studies do not compare the MS population to controls, thereby failing to provide relative results. Even when comparisons are made, they are often unable to adjust for a range of variables (e.g., ethnicity and geographical region), which limits their ability to control for potential confounding factors that might affect the accuracy and interpretation of the results. Adjusting for ethnicity is important as the general population in Denmark includes 12% immigrants and descendants compared to 0.8% in the Danish MS population. Immigrants and descendants may have different characteristics such as socioeconomic factors like educational level, income, and family structure influencing the risk of comorbidities, but also a different susceptibility for MS and comorbidities in general (51). A study investigating comorbidities found a higher prevalence among immigrants compared to long-term residents underlining the importance of taking ethnicity into account when investigating the consequences of MS (52).

Healthcare data from Denmark benefits uniquely from the Danish universal healthcare system, which provides free and equal services to all citizens regardless of income, effectively reducing the potential for bias arising from high costs. This effectively minimizes potential biases that could distort healthcare data in countries with substantially different healthcare systems.

### 4.4 Limitations

First, the cross-sectional design used in parts of the study does not allow for the establishment of causal relationships between variables and does not take temporal changes into account. As a result of this, we are not able to project the future trajectory of the observed differences between people with MS and the general population. Second, all comparisons between the two groups are univariate, and thus unadjusted for potential confounders apart from the matching covariates. Third, in the Cox regression, we assumed a non-informative censoring mechanism for emigration. However, one could argue, that individuals able to emigrate indicate a better health status than the average health status of the study population, which could lead to an overestimation of death rates. However, only 0.37 in the general population and 0.16 in the MS population did emigrate, and we consider this potential bias negligible. Fourth, due to the complexity of recent treatment trajectories, we did not include exposure to different DMTs. Finally, our study did not adjust for differences in lifestyle factors such as smoking and alcohol consumption, which could contribute to all-cause mortality. The primary objective of our investigation was to compare health outcomes between individuals with MS and a control group from the general population. The study design was intentionally broad and not equipped to isolate the potential contributory factors to mortality or to dissect the direct impacts of MS vs. other variables not included in the matching process. We aimed to provide an epidemiological overview highlighting the disparities in all-cause mortality in relation to the burden of chronic comorbidities, which are inherently influenced by a variety of factors. While our matching strategy was designed to control for the most substantial known confounders, we recognize that it is not possible to account for all potential confounding factors in observational research.

## 5 Conclusion

This study demonstrates a higher occurrence of both acute and chronic comorbidities in people with MS, as well as how comorbidities increase the hazard of death among the studied individuals. Therefore, our results underscore the importance of considering comorbidities when treating people with MS.

## Data availability statement

The datasets presented in this article are not readily available because access to the used data is only available upon qualified request and approval by the Knowledge Center for Data Reviews (entity responsible for data of the Capital Region of Denmark, approved by the Danish Data Protection Agency) and the Danish Multiple Sclerosis Group (DMSR). Requests to access the datasets should be directed to DMSR, scleroseregisteret.rigshospitalet@regionh.dk.

## Ethics statement

Ethical approval was not required for the study involving humans in accordance with the local legislation and institutional requirements. Written informed consent to participate in this study was not required from the participants or the participants' legal guardians/next of kin in accordance with the national legislation and the institutional requirements.

## Author contributions

RH: Conceptualization, Investigation, Methodology, Visualization, Writing – original draft, Writing – review & editing. MW-H: Data curation, Formal analysis, Investigation, Writing – review & editing. FS: Writing – review & editing. MM: Resources, Supervision, Writing – review & editing.
